# The Mitogenomic Landscape of Hexacorallia Corals: Insight into Their Slow Evolution

**DOI:** 10.3390/ijms25158218

**Published:** 2024-07-27

**Authors:** Zhanfei Wei, Yang Yang, Lihui Meng, Nannan Zhang, Shanshan Liu, Liang Meng, Yang Li, Changwei Shao

**Affiliations:** 1 State Key Laboratory of Mariculture Biobreeding and Sustainable Goods, Yellow Sea Fisheries Research Institute, Chinese Academy of Fishery Sciences, Qingdao 266071, China; weizf@ysfri.ac.cn; 2 BGI-Qingdao, BGI-Shenzhen, Qingdao 266555, China; yangyang8563@stu.ouc.edu.cn (Y.Y.); zhangnannan1@genomics.cn (N.Z.); liushanshan@mgi-tech.com (S.L.); 3 College of Marine Life Sciences, Ocean University of China, Qingdao 266100, China; 4 School of Marine Science and Engineering, Qingdao Agricultural University, Qingdao 266109, China; lihuimeng@qau.edu.cn; 5 Laboratory of Marine Organism Taxonomy and Phylogeny, Institute of Oceanology, Chinese Academy of Sciences, Qingdao 266071, China; liyang@qdio.ac.cn

**Keywords:** deep-sea corals, Hexacorallia, mitogenomes, phylogenetics

## Abstract

The utility of the mitochondrial genomes (mitogenomes) in analyzing the evolutionary history of animals has been proven. Five deep-sea corals (*Bathypathes* sp.1, *Bathypathes* sp.2, Schizopathidae 1, *Trissopathes* sp., and *Leiopathes* sp.) were collected in the South China Sea (SCS). Initially, the structures and collinearity of the five deep-sea coral mitogenomes were analyzed. The gene arrangements in the five deep-sea coral mitogenomes were similar to those in the order Antipatharia, which evidenced their conservation throughout evolutionary history. Additionally, to elucidate the slow evolutionary rates in Hexacorallia mitogenomes, we conducted comprehensive analyses, including examining phylogenetic relationships, performing average nucleotide identity (ANI) analysis, and assessing GC-skew dissimilarity combining five deep-sea coral mitogenomes and 522 reference Hexacorallia mitogenomes. Phylogenetic analysis using 13 conserved proteins revealed that species clustered together at the order level, and they exhibited interspersed distributions at the family level. The ANI results revealed that species had significant similarities (identity > 85%) within the same order, while species from different orders showed notable differences (identity < 80%). The investigation of the Hexacorallia mitogenomes also highlighted that the GC-skew dissimilarity was highly significant at the order level, but not as pronounced at the family level. These results might be attributed to the slow evolution rate of Hexacorallia mitogenomes and provide evidence of mitogenomic diversity. Furthermore, divergence time analysis revealed older divergence times assessed via mitogenomes compared with nuclear data, shedding light on significant evolutionary events shaping distinct orders within Hexacorallia corals. Those findings provide new insights into understanding the slow evolutionary rates of deep-sea corals in all lineages of Hexacorallia using their mitogenomes.

## 1. Introduction

Deep-sea corals thrive in environments characterized by low light and low water temperatures, including abyssal depths (up to 8600 m) [1,2,3]. In contrast with their shallow-water counterparts, deep-sea corals typically do not live a symbiotic life with unicellular algae but rely on symbiotic microbes, plankton, and organic matter from upper layers as their nutritional source [4]. Deep-sea corals play a crucial role in the nutrient-poor deep-sea environment by supporting and forming distinct, diverse, and endemic animal communities, providing precious refuge and habitats for other marine animals [1,5]. Previous studies have investigated the characteristics of deep-sea corals, including their long lifespans, slow growth rates, complex physical construction, and ecological importance within deep-sea environments [5,6,7,8,9]. A study has provided a hypothesis that the steep topography of the upper bathyal zone creates a significant environmental gradient that may drive adaptive divergence through depth differences, serving as a key source of coral biodiversity [10]. Additionally, in previous studies of Octocorallia mitogenomes, the genes encoding cytochrome b (*cyt b*) and DNA mismatch repair protein (*mutS*) may be influenced by environmental factors that affect deep-sea species formation [5]. To date, genomic research has revealed that deep-sea corals experience distinct selection pressures and have a slower evolution rate compared to their shallow-water counterparts [10,11].

The subclass Hexacorallia encompasses six orders (Actiniaria, Antipatharia, Ceriantharia, Corallimorpharia, Scleractinia, and Zoantharia) and comprises a total of 198 recorded families, which are widely distributed across various marine environments [12,13,14,15,16,17]. Deep-sea corals include various species from the subclass Hexacorallia, such as black corals (Antipatharia) and stony corals (Scleractinia). Among them, the species in the order Antipatharia are known for their ability to form proteinaceous axial skeletons, while those in the order Scleractinia develop calcareous exoskeletons [14]. The diversification of hybridization processes has facilitated the emergence of numerous genera and generated the highest species richness within the order Scleractinia, allowing Scleractinia corals to occupy a wide range of sea depths from the intertidal zone to depths of over 6000 m [10,18].

The haploid nature and uniparental inheritance of metazoan mitochondrial DNA result in an effective population size four times smaller than that of nuclear DNA and leads to faster lineage sorting [11,19,20]. Due to their relatively high substitution rate compared with nuclear genomes, metazoan mitogenomes are commonly employed in studying phylogenetic relationships and genetic diversity [21]. The coral mitogenomes exhibit typical features observed in Cnidarians, including a circular form and the presence of 13 protein-coding genes (PCGs) involved in oxidative phosphorylation and responsible for energy anabolism, in addition to two ribosomal RNA (rRNA) genes [5,21,22]. Although under strong functional constraints, mitochondrial DNA is thought to evolve under purifying selection in response to harsh environmental pressures [23]. Additionally, the mitogenomes remain an invaluable tool for examining the evolutionary relationships and genetic diversity of corals and other organisms [24]. The 13 PCGs have been extensively utilized in phylogenetic and population genetics studies, especially the *cox1* gene [19,25].

In this study, five mitogenomes of deep-sea corals within Antipatharia from the South China Sea (SCS) were sequenced and annotated at depths from 430 m to 1253 m. Initially, the structures and collinearity of the five deep-sea coral mitogenomes were analyzed. Subsequently, 522 reference Hexacorallia mitogenomes from the NCBI combined with five deep-sea coral mitogenomes were utilized to investigate their slow evolutionary rates through various analyses, including construction of phylogenetic trees, GC-skew dissimilarity assessment, and average nucleotide identity (ANI) comparisons. Additionally, 49 Hexacorallia mitogenomes from 49 different families were used to elucidate their evolutionary timelines. Through these results, we not only added to the availability of deep-sea coral mitogenomes but also proved that the mitogenomes were useful tools to verify the slow evolutionary rates of Hexacorallia corals.

## 2. Results and Discussion

### 2.1. Deep-Sea Coral Diversity and Adaptation Study

**Figure 1:**
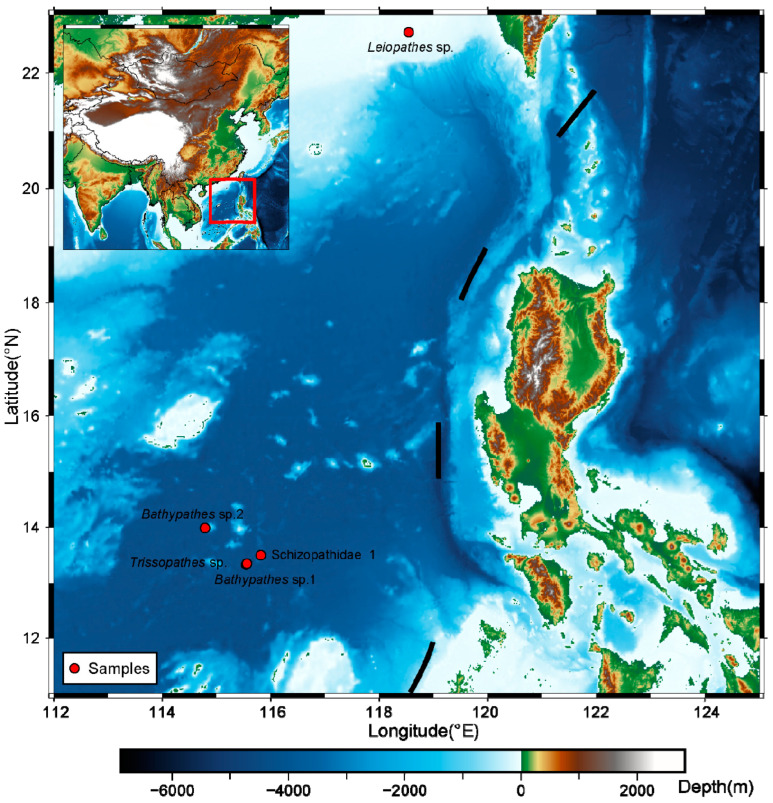
The sites of the five deep-sea corals collected in the SCS. Generic Mapping Tools (GMT6) (v6.2.0) was used to draw the sampling locations [28]. Red dots represent the sampling locations. The colors in the legend denote different depths. Detailed information about the samples is shown in Table 1.

Five deep-sea corals were sampled by the manned submersible Shenhai Yongshi from the SCS at depths ranging from 430 m to 1253 m in November 2020 (Figure 1 and Table 1). Among the deep-sea corals, four were retrieved from the bathypelagic zone (depth > 1000 m), while the remaining sample was collected from the mesopelagic zone (depth between 200 m and 1000 m) (Figure 1 and Table 1). Notably, significant variations in environmental factors were observed, with a water depth of 1000 m serving as the demarcation point [26,27]. Temperature gradients revealed that the water temperature exceeded 9 °C at ~400 m depth, whereas it dropped below 4 °C at around 1000 m depth (Table 1). The salinity measurements indicated values of 34.50 PSU for deep-sea water, contrasting with values of 34.33 PSU for shallow water (Table 1). Moreover, disparities in the seawater density were observed, with a greater density occurring in the deep sea (Table 1).

These deep-sea corals had their skeletal structures predominantly composed of chitin (Supplementary Figure S1), which was also observed in other Hexacorallia corals [29,30]. Chitin, as a vital protective and supportive element, can offer protection and support to the soft bodies of organisms, which has been demonstrated in various species, including crustaceans, tube worms, and marine sponges, in the deep-sea environment [31,32,33]. In black corals, the integration of chitin with other components and quinonic pigments can reinforce the elastic properties of the skeletal structure [29]. Therefore, chitin might serve as a protective strategy to help deep-sea corals resist the high hydrostatic pressure and maintain their structural integrity in the deep-sea environment.

### 2.2. Mitogenomic Structure of the Deep-Sea Corals

**Figure 2:**
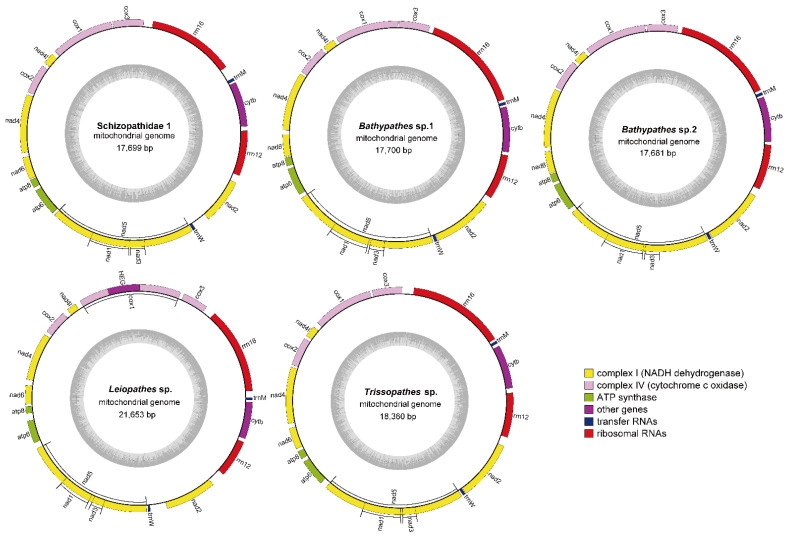
The circular maps of five deep-sea coral species. The cycle graphs of five deep-sea coral mitogenomes were drawn on the MPI-MP CHLOROBX website (https://chlorobox.mpimp-golm.mpg.de/OGDraw.html (accessed on 9 July 2024)) using the Organellar Genome DRAW (OGDRAW) function [34]. The gene maps showed the arrangements and the positions of the 13 PCGs, two rRNA genes, and two tRNA genes. Pink columns: cytochrome c oxidase genes; yellow columns: NADH dehydrogenase genes; green columns: ATP synthase genes; purple columns: cytochrome b gene; red columns: ribosomal RNA genes; dark blue columns: transfer RNA genes.

The mitogenome size and GC content of the five deep-sea corals ranged from 17,681 (*Bathypathes* sp.2) to 21,653 (*Leiopathes* sp.) bp and from 0.39% (*Leiopathes* sp. and *Trissopathes* sp.) to 0.40% (Schizopathidae 1, *Bathypathes* sp.1 and *Bathypathes* sp.2) (Table 1). The significant variation in size among five deep-sea coral mitogenomes was possible due to differences in the length of intergenic regions and the presence of a homing endonuclease gene (HEG) within the cytochrome c oxidase subunit 1 (*cox1*) intron, which was consistent with findings in black corals [13]. All these five deep-sea coral mitogenomes contained 13 PCGs, two rRNA genes (*rrn12* and *rrn16*), and two tRNA genes (*trnW* and *trnM*) on the positive-sense strand (Figure 2). The PCGs encoded *cox1*, cytochrome c oxidase subunit 2 (*cox2*), cytochrome c oxidase subunit 3 (*cox3*), NADH dehydrogenase subunit 1 (*nad1*), NADH dehydrogenase subunit 2 (*nad2*), NADH dehydrogenase subunit 3 (*nad3*), NADH dehydrogenase subunit 4 (*nad4*), NADH dehydrogenase subunit 4l (*nad4l*), NADH dehydrogenase subunit 5 (*nad5*), NADH dehydrogenase subunit 6 (*nad6*), ATP synthase F0 subunit 6 (*atp6*), ATP synthase F0 subunit 8 (*atp8*), and *cyt b*.

Interestingly, *Leiopathes* sp. had one HEG inserted in the intron of the *cox1* gene, which phenomenon was also observed in other coral mitogenomes. The insertion has been found in five out of seven families within the order Antipatharia, as well as in most species of sea anemones, Scleractinia, and Corallimorpharia [13,35,36]. The presence of the HEG is speculated to result from horizontal gene transfer, where genetic material is transferred between different species at a high rate due to the close association between introns and HEG [37,38,39]. A modified Goddard-Burt cyclic model explains the invasion, persistence, and degradation of HEGs in corals [40]. Hence, the presence of the HEG in *Leiopathes* sp. might be attributed to the genomic arrangement events.

Gene rearrangements in invertebrate mitogenomes are relatively common compared with most vertebrate mitogenomes, yet they remain unique and rare events that are unlikely to occur independently across different evolutionary lineages [41,42]. Therefore, the gene order of the complete mitogenomes can effectively infer relationships within Metazoan species [43,44]. Nine gene arrangement patterns reported in subclass Hexacorallia may indicate evolutionary relationships, functional adaptations, and key transitional states in coral evolution [45,46]. These gene arrangements suggest both minor and major genomic rearrangements, with extensive rearrangements noted in order Corallimorpharia and relatively fewer changes observed in the order Scleractinia [44]. In the study, only one gene order (*cyt b*, *rrn12*, *nad2*, *trnW*, and *nad5* inserted by *nad3* and *nad1*, *atp6*, *atp8*, *nad6*, *nad4, cox2*, *nad4l*, *cox1*, *cox3*, *rrn16*, and *trnM*), was found (Figure 2). Collinearity analysis was also employed to enhance clarity regarding the relative gene arrangements (Supplementary Figure S2). Those results further supported the observation of fewer genomic changes within Scleractinia [44]. Additionally, insertions of both the *nad3* and *nad1* genes within the *nad5* intron region were observed in five deep-sea coral mitogenomes, which indicated that rearrangement and recombination events were present in the evolution of their mitogenomes [47].

### 2.3. Evolutionary Status of the Five Deep-Sea Corals

**Figure 3:**
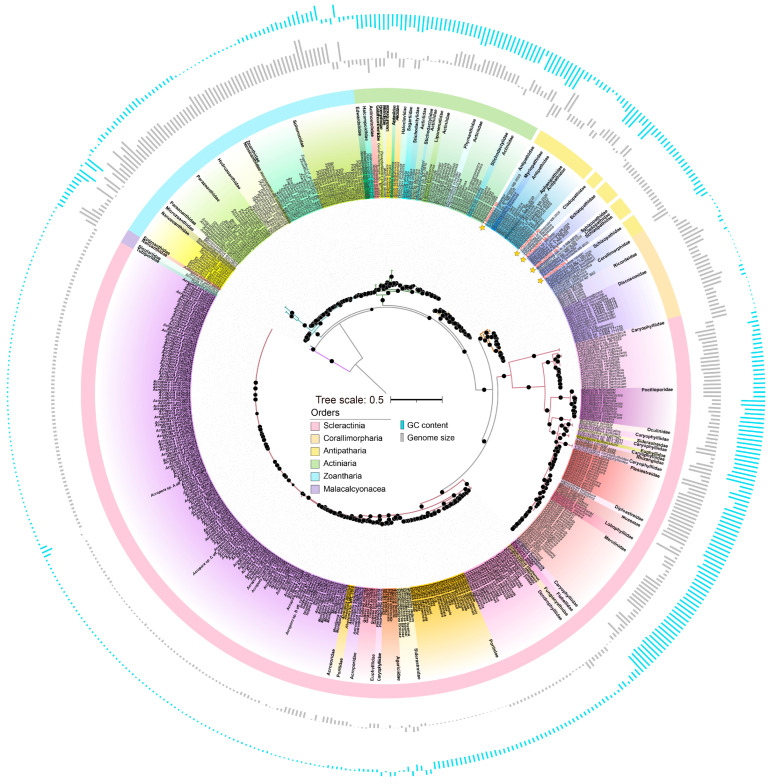
Maximum likelihood phylogenetic tree of Hexacorallia mitogenomes. The tree was constructed based on the concatenated proteins of the 13 PCGs from 530 coral species by iq-tree (v1.6.12) software [53], among which Octocorallia mitogenomes were used as the outgroup. Five deep-sea corals were marked with yellow stars in this study. The colors in the phylogenetic tree represent 49 families within Hexacorallia. The concentric cycles from inter to outer were the orders, genome sizes, and GC contents. The average genome size (18,732 bp) and GC content (38.60%) were used as baseline values in each cycle. The mitogenomes were listed in Supplementary Table S1.

A phylogenetic tree was constructed for the 13 conserved proteins to evaluate the evolutionary status of five deep-sea corals in conjunction with 522 Hexacorallia corals and Octocorallia corals as an outgroup (Figure 3). It was worth noting that those five deep-sea corals were classified within the order Antipatharia.h *Leiopathes* sp. and *Leiopathes expansa* were found to be closely related, both belonging to the family Leiopathidae. Similarly, *Bathypathes* sp.1 and *Bathypathes* sp.2 exhibited a sibling relationship with *Bathypathes* sp. n. 2 NB-2020 and *Telopathes* sp. NB-2020, respectively, from the family Schizopathidae. Schizopathidae 1 was closely related to *Parantipathes* sp. USNM 1404491, belonging to family Schizopathidae. Additionally, *Trissopathes* sp. was found to be a sister species of *Trissopathes* cf. *tetracrada* NB-2020, within the family Cladopathidae. Additionally, species within the same order were clustered together while showing interspersed distributions at the family and genus levels, which reflected complex pattern of evolutionary relationships within Hexacorallia. The slow evolution of mitogenomes might result in the interspersed distribution of species from different families or genera in the phylogenetic tree. This genetic conservation could cause unrelated species to cluster together due to shared ancestral mitochondrial sequences.

The *cox1* protein sequences of the total 530 coral mitogenomes were also utilized to gather evolutionary information. The results differed from the inferred evolutionary statuses derived from the phylogenetic analysis based on the 13 PCGs (Supplementary Figure S3). The *cox1* gene has commonly been employed in phylogenetic analysis for various species [48]. However, it has also been observed that this gene exhibits limited divergence among species within the same genus, rendering it ineffective in distinguishing their relationships [19,25]. Specifically, in the case of cnidarians, the *cox1* gene is inadequate to facilitate straightforward diagnosis [25].

These findings aligned with previous studies indicating the utility of mitochondrial sequences in macro-evolutionary investigations of cnidarians, while acknowledging the challenges associated with reconstructing phylogenetic and phylogeographical relationships between precise taxa [49,50]. Corals in the order Scleractinia are notable for their significant habitat heterogeneity, occurring in tropical and subtropical seas, as well as in the Antarctic and Arctic regions, and inhabiting both shallow and deep-sea environments, which could lead to diverse ecological adaptations [10,51]. Conversely, Corallimorpharia and Antipatharia are primarily found in deep-water environments, while Actiniaria tend to predominantly reside in shallow waters [52]. Previous studies also verified that environmental factors might drive the formation of deep-sea coral species [5]. Despite coral mitogenomes having extremely slow mutation rates, it could be speculated that within a specific clade, their diverse living conditions might contribute to obtaining valid phylogenetic signals. Hence, it could be hypothesized that the phylogenetic distance derived from mitogenomes, in combination with morphological identification, might serve as a crucial tool for the taxonomic identification of deep-sea corals.

### 2.4. GC-Skew Dissimilarity and ANI Analyses in Hexacorallia Corals

**Figure 4:**
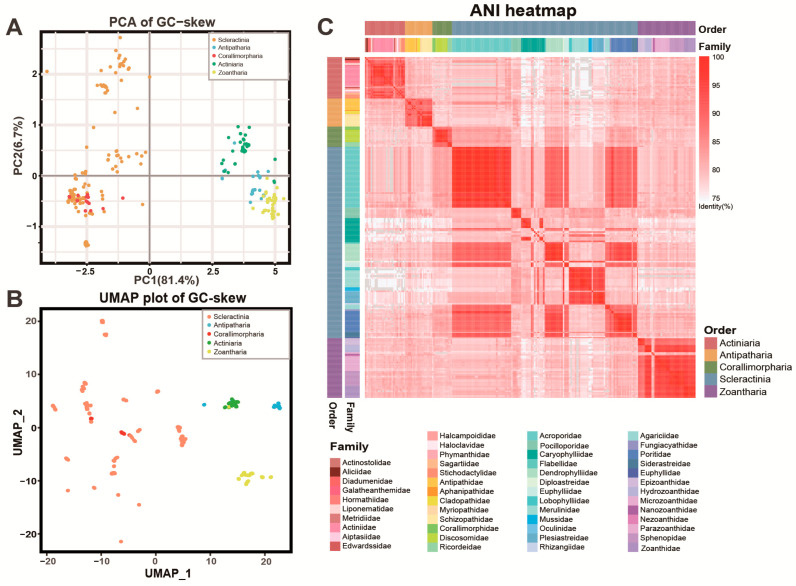
The PCA and UMAP analysis of GC-skew dissimilarity and ANI plotting of Hexacorallia mitogenomes. The GC-skew and ANI were computed by using the R script (v4.0.3) and fastANI software, respectively, to differentiate between various groups of Hexacorallia corals based on their mitogenomic structures. The results were visualized by R scripts (v4.0.3). (**A**,**B**) 13 PCGs from five deep-sea corals and reference Hexacorallia corals were utilized for the computation of GC-skew, followed by dimension reduction using PCA and UMAP analysis at the order level. (**C**) The ANI values were calculated based on pairwise comparisons of the Hexacorallia mitogenomes, which were subsequently sorted according to their existing taxonomy. The annotations provided represent orders and families. The mitogenomes were listed in Supplementary Table S1.

Previous studies indicated that variations in nucleotide frequencies in animal mitogenomes were influenced by replication events and asymmetric mutations, which might potentially be the primary sources of GC-skew variations [54,55,56]. Through the analysis of the GC-skew dissimilarity of the PCGs, insights into the structural disparities in the Hexacorallia mitogenomes were obtained, highlighting distinct mutational profiles within coral populations (Figure 4A,B and Supplementary Figure S4). The GC-skew dissimilarities for each PCG were assessed using principal component analysis (PCA) and uniform manifold approximation and projection analysis (UMAP), where each point represented an individual coral mitogenome. These results revealed that the GC-skew dissimilarity was highly significant at the order level, except for Scleractinia and Corallimorpharia (Figure 4A,B), but not as pronounced at the family level (Supplementary Figure S4). Actiniaria, Antipatharia, and Zoantharia formed distinct clusters, while Corallimorpharia interspersed with Scleractinia, consistent with their evolutionary relationships (Figure 3 and Figure 4). The notably broader dispersion range of points within the order Scleractinia hinted at higher species diversity and divergence, possibly due to significant habitat heterogeneity [10,51]. The ANI values revealed notable similarities (identity > 85%) within the same order but notable differences between different orders (identity < 80%) (Figure 4C). Particularly, significant variations in ANI values were observed between families within the order Scleractinia, which might contribute to their evolutionary diversity [10,51]. The wide geographical distribution of Scleractinia might reflect their molecular diversity, especially in their mitogenomes that encode crucial metabolic functions for survival under extreme conditions [57]. Therefore, the extensive range of GC-skew and significant variations in ANI observed among Scleractinia species suggested that the evolution of their mitogenomes might be influenced by their diverse habitats.

### 2.5. Time-Calibrated Phylogenetics of Hexacorallia Corals

**Figure 5:**
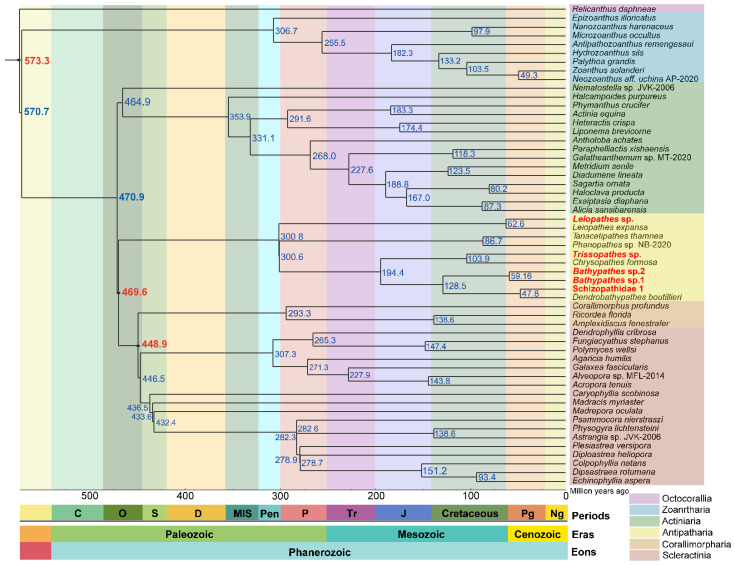
Divergence times of Hexacorallia corals at family level based on their mitogenomes. The divergence time points of the 49 families within Hexacorallia were calculated based on 13 PCGs of the mitogenome. Each species represents one family. The three red circles represent the fossil times used to calibrate the molecular clock. The columns below the tree are the geologic ages. Eon: Phanerozoic; eras: Paleozoic Era, Mesozoic Era, and Cenozoic Era; periods: O (Ordovician Period), S (Silurian Period), D (Devonian Period), MIS (Mississippian Period), Pen (Pennsylvanian Period), P (Permian Period), Tr (Triassic Period), J (Jurassic Period), Cretaceous Period, Pg (Paleogene Period), and Ng (Neogene Period). The mitogenomes were listed in Supplementary Table S2.

To elucidate the evolutionary history of Hexacorallia corals, 49 Hexacorallia mitogenomes representing 49 families combined with five deep-sea coral mitogenomes were utilized to estimate their family-level divergence times (Figure 5). The initial divergence time of corals into two major subclasses occurred ~573.3 million years ago (Ma) during the Precambrian, which was slightly older than the divergence time (~549 Ma; CI 540–574 Ma) estimated by the genomic data [58]. Those results dated the divergence of the order Scleractinia to ~446.5 Ma (95% CI 441–455 Ma) during the Ordovician epoch, which was older than the previously estimated earliest appearance of ~425 Ma at the boundary between the Ordovician and Silurian periods [58]. The divergence time estimates varied due to differences in the genetic data (nuclear genomes or mitochondrial genomes), computational methods (e.g., maximum likelihood and Bayesian inference), and fossil calibration strategies [15,58,59]. The discrepancy in the divergence times emphasized the necessity of integrating more methods to accurately decipher evolutionary dynamics of Hexacorallia. Additionally, these estimations stemmed from meticulous fossil-calibrated phylogenetic analysis, enhancing understanding of the evolutionary timelines within these coral groups.

## 3. Materials and Methods

### 3.1. Ethical Statement

Ethical approval for all deep-sea coral experiments was obtained from the Yellow Sea Fisheries Research Institute, the Chinese Academy of Fishery Sciences, in accordance with the guidelines outlined in the Regulations for the Administration of Affairs Concerning Experimental Animals of China (Qingdao, China) (approval no.: YSFRI-2024048).

### 3.2. Sample Acquisition and Treatment

During the November 2020 expedition aboard the research vessel Tan Suo Yi Hao, five deep-sea coral samples were collected from depths ranging from 430 m to 1253 m using the manned submersible Shenhai Yongshi with mechanical arms during five dives and then stored in a bio-box (Figure 1 and Table 1). Once the submersible resurfaced, these deep-sea coral samples were washed with deionized water and retained at −80 °C in the onboard laboratory. The coral specimens were immersed in liquid nitrogen and then ground using sterilized mortars in the laboratory. To extract total genomic DNA, we employed the phenol–chloroform method with modifications, as described in previous studies [5]. The quality and quantity of the extracted DNA were assessed using gel electrophoresis and a Qubit 2.0 Fluorometer (Life, Carlsbad, CA, USA). An amount of 1 µg of DNA was fragmented to 500 bp via ultrasonication, and then the DNA libraries were constructed using the MGIEasy Universal DNA Library Prep Kit (MGI, Qingdao, China). Subsequently, the libraries were sequenced on the MGISEQ-2000 platform, generating 100 bp paired-end reads and yielding approximately 10 Gbp per sample.

### 3.3. Genomic Assembly and Annotation

FastQC (v0.11.9) with the default settings was used to examine the raw data [60]. Subsequently, Fastp (v0.22.0) was employed to remove unqualified reads and adaptors, using the parameters ‘-f 5-F 5-w 24-c-q 20-g-W 5-3-l 50’ [61]. The filtered data were then assembled into contigs using MEGAHIT (v1.1.2) with the settings ‘--min-contig-len 1000--k-list 21, 33, 55, 77, 99, 127’ [62]. A total of 522 reference Hexacorallia mitogenomes retrieved from the NCBI (number of PCGs > 10 and mitogenome length > 10 kbp) (Supplementary Table S1) were utilized to search for potential mitochondrial contigs using Blastn. The mitochondrial potential reads were extracted using Bowtie2 (v2.4.4) with the ‘--very-fast-local’ option, enabling a refined comparison with the quality-filtered data. Finally, the potential reads were reassembled using complete SPAdes (v3.6.2) with the parameters ‘--careful-k 21,33,55,77,99’ to obtain the mitogenomes [63].

The primary annotation of the five deep-sea coral mitogenomes was performed by the Ge-SEQ webservers (https://chlorobox.mpimp-golm.mpg.de/geseq.html (accessed on 9 July 2024)), with the gene identity threshold set at 85% [64,65]. The online software tRNAscan-SE2.0 (http://lowelab.ucsc.edu/tRNAscan-SE (accessed on 9 July 2024)) was employed to verify the accuracy of the reported number of tRNAs in the annotation results [66]. Subsequently, a meticulous manual inspection and correction of the sequences in the FASTA file and the annotation output GenBank format files were conducted, drawing on previous research on mitogenomes as a reference. The cycle graphs of the five mitogenomes were drawn on the MPI-MP CHLOROBX website (https://chlorobox.mpimp-golm.mpg.de/OGDraw.html (accessed on 9 July 2024)) using the Organellar Genome DRAW (OGDRAW) function [34].

### 3.4. Genome Structure Analyses

The ANI values were calculated through pairwise comparisons between the five deep-sea corals and 522 reference Hexacorallia mitogenomes using the software fastANI with the parameter ‘--minFraction 0.8’ [67]. Python code was employed to transform the output file from triangular matrices into a symmetric matrix format, facilitating the creation of a heatmap with in-house R scripts. Additionally, the genome sizes and GC contents of the 527 mitogenomes mentioned above were calculated using Python scripts. For this purpose, all the protein-coding gene (PCG) sequences from each genome were extracted to calculate the GC-skew values (GC-skew = (G − C)/(G + C)). The PCA analysis of the GC-skew dissimilarity (method = Euclidean) was conducted with the vegdist function in R (v4.0.3). Additionally, the UMAP analysis for the dimensional reduction of the GC-skew values was performed using the in-house script in R (v4.0.3) as well. In addition, mitogenome collinearity analysis was carried out to assess the distribution or arrangement of homologous genes within or across the five deep-sea corals using JCVI [68].

### 3.5. Phylogenetic Analyses

To delineate the phylogenetic relationships between five deep-sea coral mitogenomes, we combined them with 522 reference Hexacorallia mitogenomes and reference Octocorallia mitogenomes as the outgroup. Each protein-coding gene from all mitogenomes was individually extracted and then aligned using MAFFT L-INS-I (v7.294b) [69]. These sequences were trimmed to remove poorly aligned regions by employing the trimAl (v1.4) software with the parameter ‘-automated1’, respectively [70]. These trimmed PCGs, which were from the same mitogenome, were joined together and placed into one FASTA file using in-house scripts and then were used to construct the phylogenetic tree using the iq-tree (v1.6.12) (‘-m MFP-bb 1000-alrt 1000’) [53]. Additionally, the *cox1* protein sequence was extracted from all mitogenomes and aligned by ClustalW in MEGA-X, followed by the construction of a neighbor-joining (NJ) tree in MEGA-X with these aligned sequences.

### 3.6. Time Estimation in Phylogenetic Analysis

To estimate the evolutionary timelines, 49 Hexacorallia mitogenomes representing 49 families along with five deep-sea coral mitogenomes were used to construct a primary phylogenetic tree based on the 13 PCGs sequences by iq-tree (v1.6.12) (‘-m MFP-bb 1000-alrt 1000’) [53]. To calibrate the molecular clock, three fossil data (Octocorallia and Hexacorallia: 406.0–635.8 Ma; Antipatharia, Corallimorpl, and Scleractinia: 470.5 Ma; Corallimorpl and Scleractinia: 263.1–451.7 Ma) were utilized using TimeTree (http://www.timetree.org/ (accessed on 9 July 2024)) [71]. Furthermore, these time points were incorporated into the prepared tree file, and the divergence times were calculated using the mcmctree function in PAML (v4.9) [72]. The resulting timelines were correlated with the geological time scales, providing a detailed view of evolutionary histories in alignment with the Earth’s geologic history.

## 4. Conclusions

Deep-sea corals play a vital role in sustaining the deep-sea ecosystem. They can form long-lived reefs and live in deep oceans with a relatively stable environment compared to shallow water. Relevant research in this field holds both ecological and scientific significance. We collected five deep-sea corals at depths ranging from 430 m to 1253 m during one cruise and focused on exploring the structural characteristics of their mitogenomes. Through the analysis of 522 reference mitogenomes of Hexacorallia combined with five deep-sea corals, we investigated their evolutionary status including the variances in GC-skew, ANI, and mitogenomic structure at the order and family levels, which revealed the slow evolutionary rates in the Hexacorallia mitogenomes. Additionally, study also underscored the importance of integrating mitochondrial and nuclear genomic data to accurately decipher the divergence time of Hexacorallia, particularly as mitogenomes tend to show older divergence times due to their slow evolutionary rate.

## Figures and Tables

**Table 1:** Information on mitogenomes of five deep-sea corals.

Name	Genome Size (bp)	GC Content (%)	Depth (m)	T (°C)	Salinity (PSU)	Density (kg/m^3^)	Longitude (°E)	Latitude (°N)
*Leiopathes* sp.	21,653	0.39	430 m	9.28	34.33	1028.50	118.54	22.70
Schizopathidae 1	17,699	0.40	1105 m	3.90	34.48	1032.48	115.81	13.50
*Bathypathes* sp.2	17,681	0.40	1176 m	3.70	34.50	1032.85	114.79	13.99
*Trissopathes* sp.	18,360	0.39	1180 m	3.91	34.48	1032.82	115.56	13.35
*Bathypathes* sp.1	17,700	0.40	1253 m	3.70	34.49	1033.18	115.54	13.33

## Data Availability

The genome sequence data of this study are available in GenBank of the NCBI (https://www.ncbi.nlm.nih.gov/ (accessed on 9 July 2024)), with accession numbers OR906317, OR906318, OR992049, OR992050, and OR992051 representing each deep-sea coral.

## Supplementary Materials

The following supporting information can be downloaded at: https://www.mdpi.com/article/10.3390/ijms25158218/s1. Supplementary Figures S1–S4 and Supplementary Tables S1 and S2.
