# Supplementary material for: The Mitogenomic Landscape of Hexacorallia Corals: Insight into Their Slow Evolution
Source: Int J Mol Sci. 2024 Jul 27;25(15):8218. doi: 10.3390/ijms25158218 (PMC11311739; doi:10.3390/ijms25158218)
Supplement: Supplementary file 1 [file ijms-25-08218-s001.zip › Supplementary Figure S4.pdf]

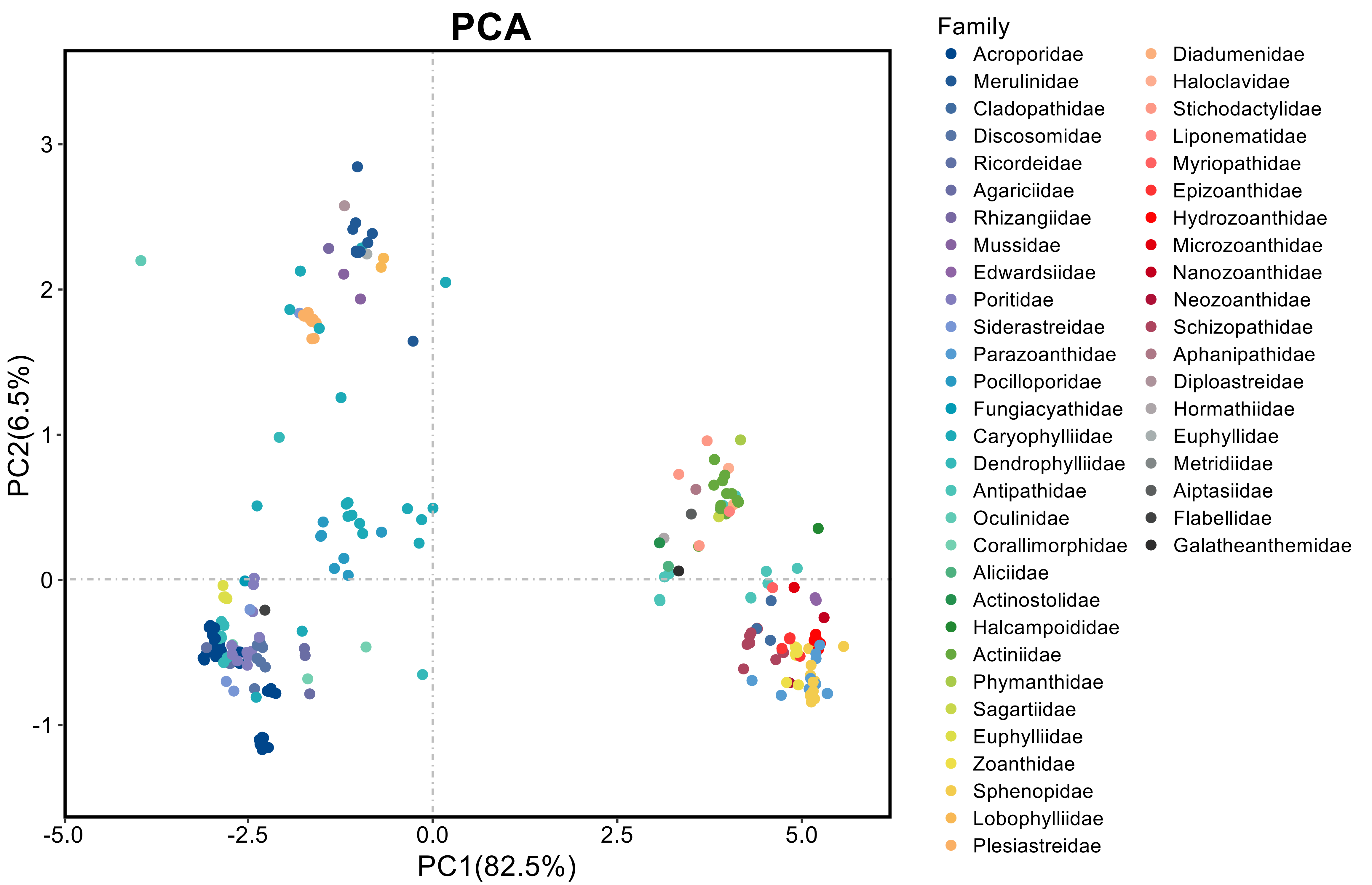
PCA
PC2(6.5%)
PC1(82.5%)
3
2
1
0
-1
-5.0
-2.5
0.0
2.5
5.0
Family
Acroporidae
Merulinidae
Cladopathidae
Discosomidae
Ricordeidae
Agariciidae
Rhizangiidae
Mussidae
Edwardsiidae
Poritidae
Siderastreidae
Parazoanthidae
Pocilloporidae
Fungiacyathidae
Caryophylliidae
Dendrophylliidae
Antipathidae
Oculinidae
Corallimorphidae
Aliciidae
Actinostolidae
Halcampoididae
Actiniidae
Phymanthidae
Sagartiidae
Euphylliidae
Zoanthidae
Sphenopidae
Lobophylliidae
Plesiastreidae
Diadumenidae
Haloclavidae
Stichodactylidae
Liponematidae
Myriopathidae
Epizoanthidae
Hydrozoanthidae
Microzoanthidae
Nanozoanthidae
Neozoanthidae
Schizopathidae
Aphanipathidae
Diploastreidae
Hormathiidae
Euphyllidae
Metridiidae
Aiptasiidae
Flabellidae
Galatheanthemidae
