# Supplementary figures and images for: The Mitogenomic Landscape of Hexacorallia Corals: Insight into Their Slow Evolution
Source: Int J Mol Sci. 2024 Jul 27;25(15):8218. doi: 10.3390/ijms25158218 (PMC11311739; doi:10.3390/ijms25158218)

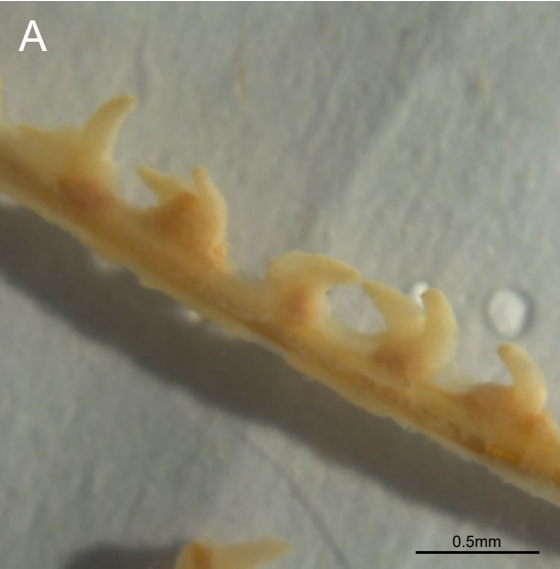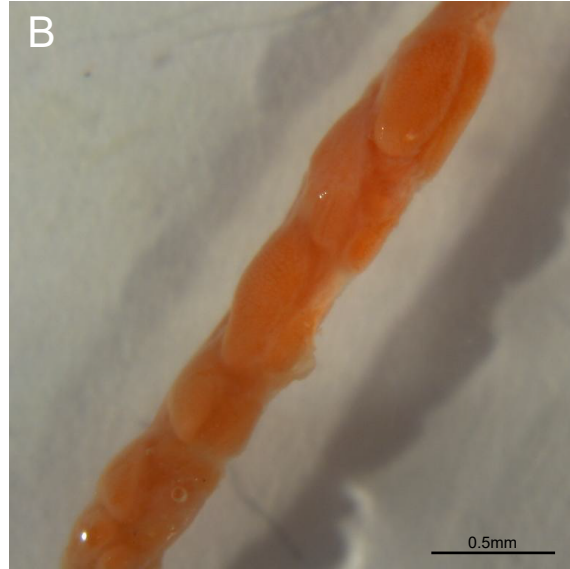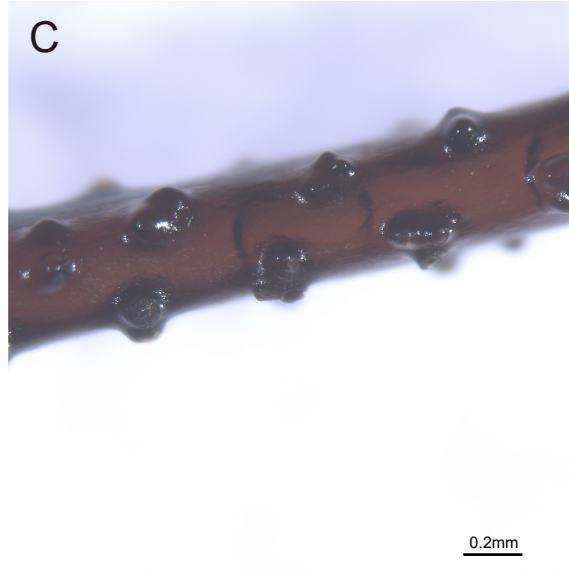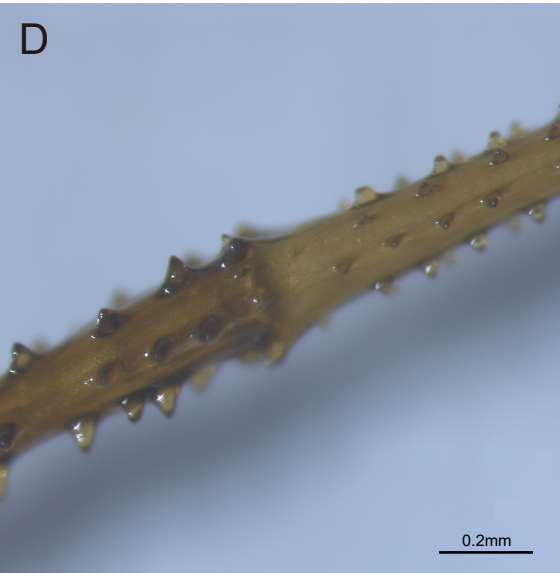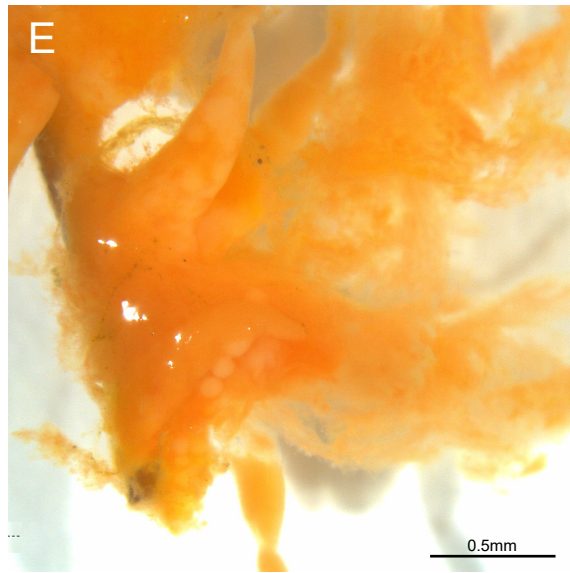

A: *Trissopathes* sp.

B: *Bathypathes* sp.1

C: *Leiopathes* sp.

D: Schizopathidae 1

E: *Bathypathes* sp.2

Supplement: Supplementary file 1 [file ijms-25-08218-s001.zip › Supplementary Figure S1.pdf]

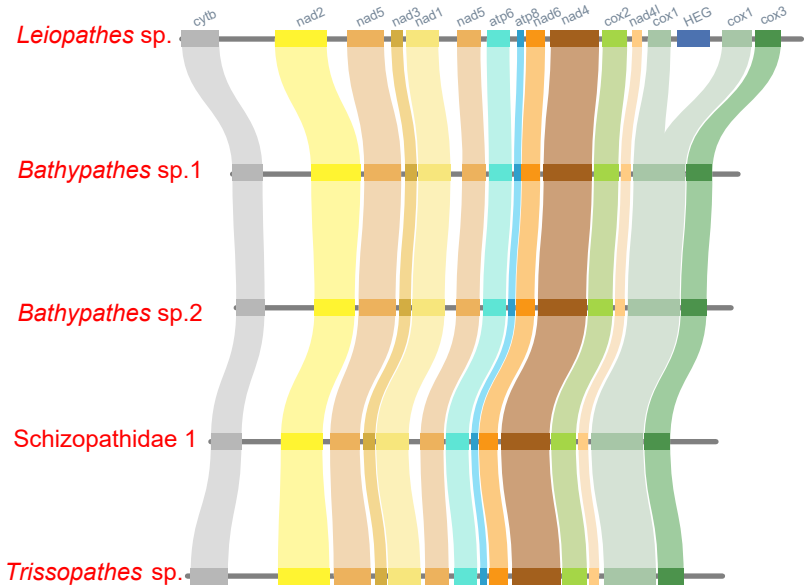

Supplement: Supplementary file 1 [file ijms-25-08218-s001.zip › Supplementary Figure S2.pdf]

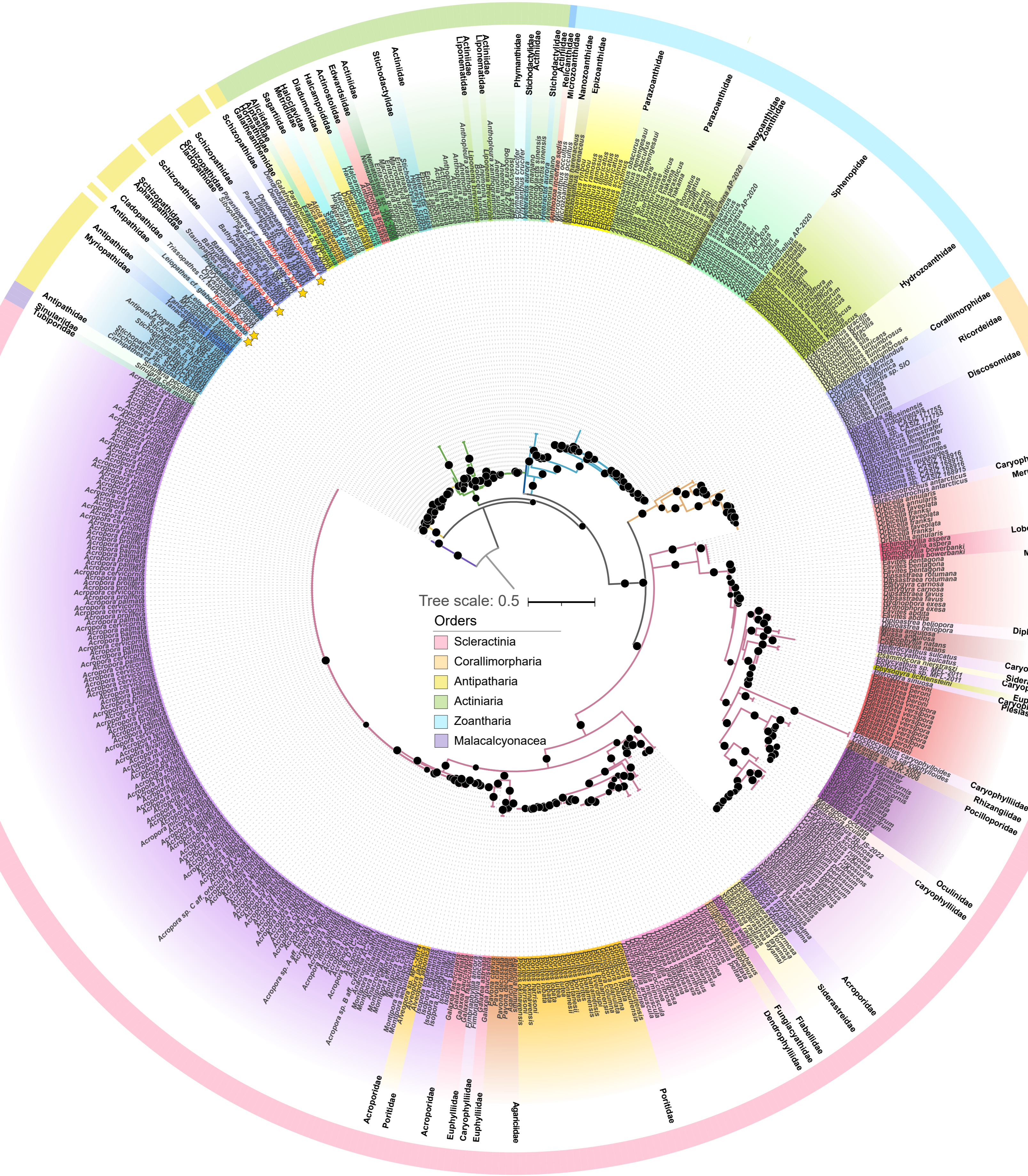

Supplement: Supplementary file 1 [file ijms-25-08218-s001.zip › Supplementary Figure S3.pdf]
